# Intensity Noise Suppression in Photonic Detector Systems for Spectroscopic Applications

**DOI:** 10.3390/s25226932

**Published:** 2025-11-13

**Authors:** Yupeng Wu, Kai Ma, Zhou Wu, Wenxi Zhang

**Affiliations:** 1 Aerospace Information Research Institute, Chinese Academy of Sciences, Beijing 100094, China; wuyupeng22@mails.ucas.ac.cn (Y.W.); makai1207@yeah.net (K.M.); wz@aircas.ac.cn (Z.W.); 2 School of Optoelectronics, University of Chinese Academy of Sciences, Beijing 100049, China

**Keywords:** laser frequency stabilization, absorption spectroscopy, power noise, spectral signal sensor

## Abstract

Spectral measurement technology has found extensive applications across a diverse range of fields, including chemical analysis, environmental monitoring, precise measurement, and laser frequency stabilization. However, the accuracy of spectral measurement results is often constrained by the power noise and frequency jitter inherent in the light source. In contrast to the traditional differential amplification method for acquiring spectral signals, our study introduces a novel approach. By employing a power correction quotient, we effectively suppress common-mode noise. Additionally, we introduce a novel composite differential method that, in theory, is capable of performing closed-loop processing on spectral signals to stabilize the laser frequency. This innovative method not only constructs a stable laser source but also yields high-quality spectral signals simultaneously. In an experiment involving iodine molecule absorption spectroscopy, the algorithm we propose demonstrated remarkable efficacy in mitigating distortions caused by modulated signals and significantly enhanced the signal-to-noise ratio. This algorithm is versatile and can be applied to the signal processing of any spectral signal sensor that employs dual-path light.

## 1. Introduction

Spectral measurement technology is widely used in various applications, such as optical frequency domain reflectometry (OFDR) [1], frequency stabilization [2,3], environmental monitoring [4,5], material analysis [6], interferometry metrology, and other fields [7] due to its advantages of high precision, intuitive data presentation, and wide wavelength range.

However, the wavelength and output power of lasers are prone to severe drift. This drift originates from the material properties of the lasers, which are influenced by various factors such as current [8], temperature [9], etc. Consequently, it’s impossible to obtain a stable laser output for a free-running mode laser, which often exhibits wavelength shifts of several picometers over long periods.

For lasers, a stabilization system based on iodine molecular saturation absorption is well-known. This system leverages the inherent stability of iodine molecular natural absorption lines to achieve high long-term stability. G. R. Hanes et al. were the first to use saturated absorption spectroscopy (SAS) of iodine vapor to enhance laser stability, achieving the short-term stability of 10^−9^ and the long-term stability of 10^−10^ [10]. More recently, Shogo Matsunaga et al. proposed a numerical simulation model and utilized saturation spectroscopy to calculate the interaction-length dependence of frequency stability [3]. Despite significant advancements in laser frequency stabilization technology that have mitigated wavelength drift issues, current methods still exhibit certain limitations. Frequency stabilization methods based on molecular absorption lines offer superior long-term stability and can eliminate Doppler background noise using the third harmonic method. However, these methods often suffer from low signal intensity. Moreover, for Distributed Feedback (DFB) lasers or Distributed Bragg Reflector (DBR) lasers, electric factors such as injection current and piezoelectric transducer (PZT) voltage can cause significant changes in wavelength and power [11]. These changes are highly detrimental to sensors that rely on scanning spectroscopy to acquire information.

Consequently, the development of anti-interference algorithms has garnered significant attention. Traditional algorithms rely on frequency jitter and the phase of demodulated signals to qualitatively determine the offset frequency relative to the reference frequency. However, this approach is inherently inaccurate due to the presence of Doppler background noise. To address this limitation, demodulation algorithms based on higher harmonics were subsequently developed. These algorithms utilize third, fifth, and seventh harmonics to achieve steeper slopes [12,13]. While higher-order harmonics effectively filter out background noise, they also result in exponentially decreasing signal strength and narrower linear regions. Moreover, these algorithms are not only highly sensitive to external environmental factors but also to power noise introduced by the light source itself. Recently, a noise-immune cavity-enhanced optical heterodyne molecular spectroscopy (NICE-OHMS) algorithm has been proposed, yet it still faces certain limitations [14]. H. Xie et al. integrated visible light-infrared reflectance spectroscopy with machine learning (ML) algorithms to enable rapid detection of iron ore grades, thereby satisfying the demands of mining production [15]. Nonetheless, the model was intricate and necessitated a training dataset. W. He et al. introduced an automatic overlapping peak decomposition algorithm [16] that relies on signal smoothing, iterative sharpening, and peak fitting. However, the study did not address the power noise intrinsic to the light source itself. K. Ozawa et al. developed a local adaptive smoothing method [17], which is capable of preserving sharp peaks and reducing signal distortion. However, this method is ineffective for removing modulation noise.

In this study, we theoretically examined third-harmonic frequency stabilization technology anchored in molecular absorption lines, closely examining how power modulation noise (PMN) from the laser source and the second harmonic in the modulation signal affect the process. Drawing on this examination, we devised an anti-interference frequency stabilization signal algorithm that employs a composite differentiation method. We validated the efficacy of this algorithm via a frequency stabilization setup featuring a 633 nm semiconductor laser diode. The algorithm successfully eradicated power noise during the spectral scanning phase in the laser frequency stabilization setup, which operates on the iodine molecule absorption spectrum principle. It adeptly reconstructed the spectral signal and tripled the frequency locking range.

## 2. Methods

### 2.1. Frequency Stabilization System Based on SAS

Given that a molecular gas sample exhibits a Maxwell–Boltzmann velocity distribution, when a monochromatic laser beam at frequency ω traverses an iodine cell, the Doppler-shifted laser frequency in the moving molecular frame becomes $\omega^{\prime }=\omega \pm kv_{z}$. Significant absorption contributions from molecules occur only when this shifted frequency lies within the homogeneous broadening linewidth γ, centered at the stationary molecule’s absorption frequency $\omega_{0}$. At $\omega_{0}$, a characteristic dip, termed the Lamb dip, emerges in the absorption curve due to the reduced number of contributing particles, leading to minimal output power. Concurrently, when a weaker laser beam passes through the iodine cell in the opposite direction, Lamb peaks appear on the Doppler-broadened absorption line, thereby forming the saturation absorption spectrum. Prior research has demonstrated that incorporating saturation absorption spectra enhances spectral resolution and subsequently boosts the signal-to-noise ratio (SNR) of the frequency stabilization signal [18].

To mitigate Doppler background effects, light source modulation and frequency stabilization based on the third harmonic of the power signal are commonly employed. However, when the frequency of a semiconductor laser is modulated via current, PMN is simultaneously introduced in the output power. Moreover, the modulation signal is typically not a pure sine wave but contains higher harmonics, with the second harmonic having the most pronounced impact, thereby causing modulation harmonic noise (MHN).

**Figure 1:**
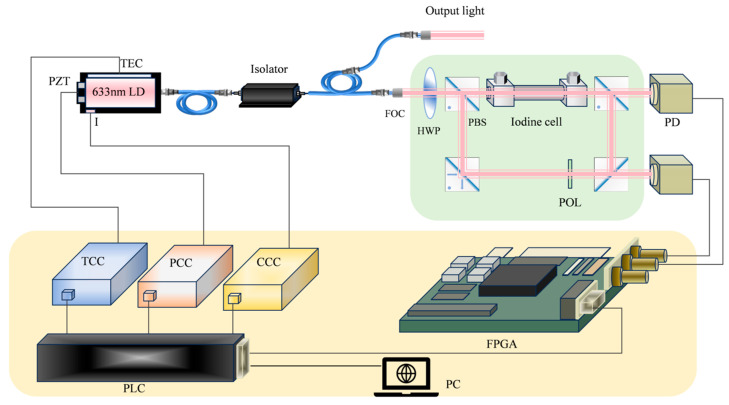
Experimental setup of a semiconductor laser at 633 nm. PCC: PZT Control Circuit; TCC: Temperature Control Circuit; CCC: Current Control Circuit.

The experimental setup for a 633 nm semiconductor laser is depicted in Figure 1. The laser emits 20 mW of power with a 1 mm beam diameter. To ensure precise alignment of the detection and saturation light within the iodine cell, the setup maintains parallelism within 0.01° and keeps eccentricity below 10 μm. The light power signal, after being amplified by detectors with an 80 kV/W gain factor, is processed by an algorithm to generate a frequency stabilization signal. To produce a differential signal and eliminate Doppler background noise, the laser current is modulated with a 10 kHz sine wave at 10 mA amplitude. To reduce environmental interference, the laser chip is equipped with dual thermoelectric coolers (TECs), maintaining temperature fluctuations below 1 mK. The laser is driven by a constant current source with 0.3 μA resolution and stability exceeding 5 ppm over 24 h. The acquisition card operates at a 10 MSa/s sampling rate. The iodine cell is kept at a constant temperature of 70 °C.

After traversing the isolator, 1% of the laser energy is diverted to the frequency stabilization system (highlighted in the green box) via a 1 × 2 optical coupler, while the remaining 99% is designated for output. The incident beam’s energy ratio between polarized and non-polarized light is then fine-tuned using a fiber optical coupler (FOC), a half-wave plate (HWP), and a polarization beam splitter (PBS). Around 20 mW of the transmitted light is employed to detect the iodine molecule absorption spectrum, while the reflected light is rerouted by another PBS. The 20 mW of reflected light that passes through the polarizer (POL) and beam splitter (BS) serves as the reference light, and the residual light is channeled back as saturated light to the iodine cells. Two photodetectors (PDs) capture the transmitted light signals, which are then collected by the Field Programmable Gate Array (FPGA) and subsequently processed by the upper computer software. The programmable logic controller (PLC) dispatches commands to the relevant actuators, which can compensate for wavelength drift.

#### 2.1.1. Laser Power Modulation Noise

For a single-mode semiconductor laser, it is assumed that both the probe light and the reference light are modulated simultaneously by a sinusoidal signal at the angular frequency $\Omega_{m}$. The transmittance α of the gas molecule absorption cell is a function of the laser wavelength. The power expressions for the probe light $P_{p}\;$ and the reference light $P_{r}$ are given as follows:

(1) $$P_{p}=\left[P_{1}+P_{1m}\sin\left(\Omega_{m}t\right)\right]\alpha \left(\lambda \left(t\right)\right),$$

(2) $$P_{r}=\left[P_{2}+P_{2m}\sin\left(\Omega_{m}t+\varphi \right)\right],$$

(3) $$\lambda \left(t\right)=\lambda_{0}+\delta \lambda \left(t\right)+\Delta \lambda \sin\left(\Omega_{m}t\right),$$

where $P_{n}(n=1,\;2)$ is the light power, $\lambda_{0}$ is the initial wavelength, *δ*(*λ*) represents the wavelength noise of the light source itself, Δλ  denotes the amplitude of the wavelength modulation signal, *t* represents time, and the phase difference φ could be corrected to 0. By calculating the Taylor expansion of the power difference signal at point $\lambda =\lambda_{0}$ and using the Prosthaphaeresis Formula, also considering that the modulation coefficient $K_{I-P}$ (current-to-power) and $K_{I-\lambda }$ (current-to-wavelength) are approximately constant, their ratio can be defined as $K_{P-\lambda }$, the ideal signal corresponding to the third harmonics can be derived as

(4) $$P_{3th,DC}=K_{P-\lambda }\left(\lambda_{0}\left(t\right)-\lambda_{th}\right)\left[\frac{{\left(\Delta \lambda \right)}^{3}}{24}{\left.\frac{d^{3}\alpha }{d\lambda^{3}}\right|}_{\lambda_{0}}-\frac{{\left(\Delta \lambda \right)}^{5}}{384}{\left.\frac{d^{5}\alpha }{d\lambda^{5}}\right|}_{\lambda_{0}}+\ldots \right]\sin\left(3\Omega_{m}t\right),$$

(5) $$P_{3th,AC}=K_{P-\lambda }\left[\frac{{\left(\Delta \lambda \right)}^{3}}{8}{\left.\frac{d^{2}\alpha }{d\lambda^{2}}\right|}_{\lambda_{0}}-\frac{5{\left(\Delta \lambda \right)}^{5}}{384}{\left.\frac{d^{4}\alpha }{d\lambda^{4}}\right|}_{\lambda_{0}}+\ldots \right]\sin\left(3\Omega_{m}t\right).$$

where $P_{3th}$ represents the third harmonic component of power, and the subscripts DC and AC indicate the direct current and alternating current, respectively, $\lambda_{th}$ is the central wavelength of the laser corresponding to the threshold current.

Clearly, the third harmonic amplitude combines the second derivative of $\alpha \left(\lambda \left(t\right)\right)$ with its third derivative. This combination is disadvantageous for feedback, as it introduces a zero-position offset that compromises the precision of frequency stabilization. Moreover, it affects the magnitude of the frequency deviation signal, resulting in feedback errors. Even under ideal conditions, random fluctuations in the light source can induce feedback errors, which are evident in the $\lambda_{0}\left(t\right)$ term. Near the reference wavelength $\lambda_{0}\left(t\right)$ the magnitudes of the zero-position offset error $\epsilon_{\lambda }$ and the feedback error $\epsilon_{P}$ can be expressed, respectively, as

(6) $$\varepsilon_{\lambda }=\frac{c}{v_{0}+10^{-4.2622}\gamma^{1.9998}}-\frac{c}{v_{0}}.$$

(7) $$\varepsilon_{P}=P_{3th,AC}\approx K_{P-\lambda }\frac{{\left(\Delta \lambda \right)}^{3}}{8}{\left.\frac{d^{2}\alpha }{d\lambda^{2}}\right|}_{\lambda_{0}},$$

where c is the speed of light, $v_{0}=\frac{c}{\lambda_{0}}$ is the frequency of the laser, γ represents laser linewidth. When $v_{0}$ is fixed, $\epsilon_{\lambda }$ only depends on γ.

**Figure 2:**
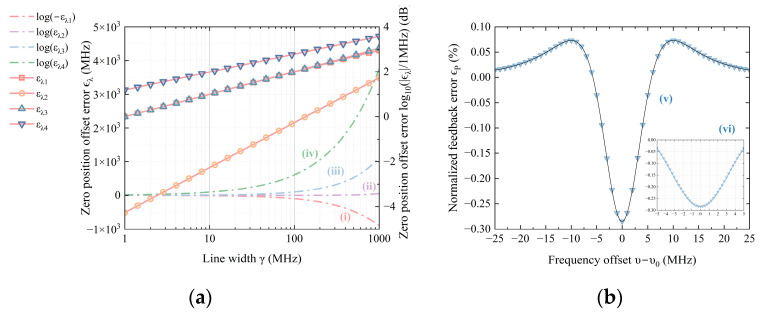
(**a**) Change in the zero-position offset error $\epsilon_{\lambda }$ as a function of the line width γ in linear coordinate (dotted line), and the absolute value of $\epsilon_{\lambda }$ in logarithmic coordinate (solid line). Curves (i), (ii), (iii), (iv) represent different real solutions of Equation (15). In logarithmic coordinates, the curves (i) and (iii) coincide. (**b**) Curves (v) is the difference between feedback signal containing PMN and ideal feedback signal @ γ = 10 MHz. Curves (vi) is a local zoom-in of the absolute value of the difference.

For a semiconductor laser with a line width on the order of MHz, and given parameters $\lambda_{0}$ = 633 nm, $\lambda_{th}$ = 633.006 nm, c = 3$\;\times \;10^{8}$ m/s, and γ = 10 MHz, the numerical solutions for $\epsilon_{\lambda }$ and $\epsilon_{P}$ as functions of $\lambda_{0}$ and γ were computed using Equations (4)–(7). The simulation results are depicted in Figure 2. To better illustrate the trends, Figure 2a employs both logarithmic (right vertical-axis) and linear (left vertical-axis) coordinates. The signal, resembling a dispersion curve with multiple zeros, yields multiple solutions. Curves (i), (ii), (iii), and (iv) represent the zero-position error. However, only the zeros near the central linear region are meaningful, making curve (ii) the most relevant. In the logarithmic coordinate, $\epsilon_{\lambda }$ clearly increases as a power function of γ with an exponent of approximately 2, determined by the least squares method. Figure 2b shows the feedback error relative to frequency at γ = 10 MHz, along with a partial enlargement. The error magnitude is an even function, positively correlated with frequency deviation. For analytical simplicity, the wavelength modulation amplitude is set to unity, and the feedback error is normalized by dividing by the peak value of the ideal signal. Near the center frequency, the feedback error reaches a maximum of −0.3%, tapering to 0 at the linear region’s edge.

#### 2.1.2. Laser Modulation Harmonic Noise

Owing to the constraints of discretization sampling and the influence of nonlinear loads, digital sinusoidal signals invariably encompass higher harmonics, with the second harmonic being the most prominent. Employing an impure sinusoidal wave for modulation infuses phase and amplitude discrepancies into the frequency stabilization signal.

The output powers incorporating the fundamental and nth harmonic waves with amplitude of *B* can be depicted as follows

(8) $$\Delta P_{har}\left(\lambda \right)=\frac{P_{0}}{2}-\frac{P_{0}}{2}\left[\alpha \left(\lambda_{0}\right)+{\left.\frac{d\alpha }{d\lambda }\right|}_{\lambda_{0}}\Delta_{n}\left(t\right)+\frac{1}{2!}{\left.\frac{d^{2}\alpha }{d\lambda^{2}}\right|}_{\lambda_{0}}\Delta_{n}^{2}\left(t\right)++\frac{1}{3!}{\left.\frac{d^{3}\alpha }{d\lambda^{3}}\right|}_{\lambda_{0}}\Delta_{n}^{3}\left(t\right)+\ldots \right],$$

(9) $$\Delta_{n}\left(t\right)=\Delta \lambda \left[\sin\left(\Omega_{m}t\right)+B\sin\left(n\Omega_{m}t\right)\right],$$

**Figure 3:**
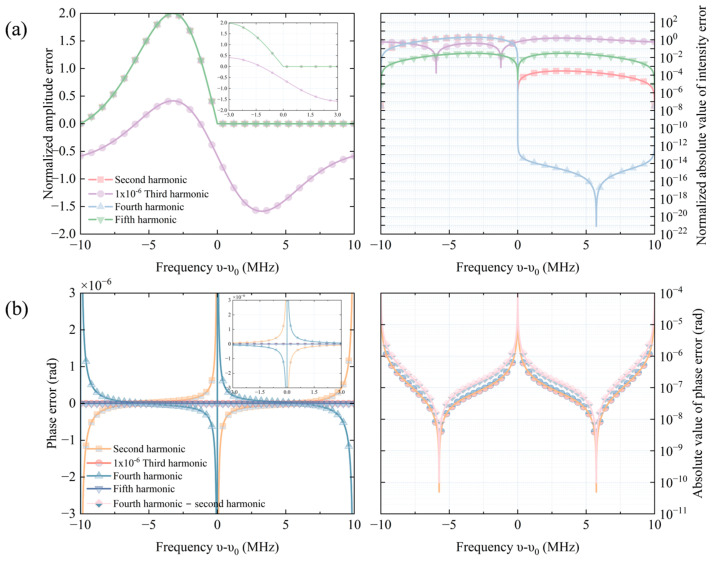
Influence of harmonics of different order on frequency stabilization signal with γ = 10 MHz. (**a**) The first line illustrates the relationship between the intensity error and the frequency deviation. It also includes a local magnification diagram and the absolute value curve of the intensity error on a logarithmic scale. (**b**) The second line depicts the relationship between the phase error and the frequency deviation. It also includes a local magnification diagram and the absolute value curve of the phase error on a logarithmic scale.

When considering only the first term of the Taylor expansion, harmonics of order higher than 5 are negligible in terms of error contribution to the solution. Figure 3 illustrates the impact of harmonics of various orders with γ = 10 MHz. Regarding amplitude error, the third harmonic has a significantly greater effect on the solution (by about six orders of magnitude) compared to other harmonics, even if its amplitude is only 1% of the fundamental wave. As depicted in Figure 3a, the influence of harmonics on signal intensity is asymmetrical. In the positive frequency deviation interval, the amplitudes of noise introduced by harmonics and the original signal are both positive. However, in the negative frequency deviation interval, the situation differs, leading to more zeros in the signal, which appear as sharp inflection points under logarithmic coordinates. For phase error, only even-order harmonics introduce additional phase errors, as shown in the partial enlargement of Figure 3b. The phase errors introduced by the second and fourth harmonics are so close that distinguishing between them requires a detailed comparison, as illustrated in Figure 3b. At the reference frequency, the phase exhibits a sharp change on the order of 10^−4^, then steeply drops to 10^−10^, and subsequently increases back to 10^−4^.

In practice, the harmonics in electrical signal will be effective depressed after passing through harmonic filter [19], so it’s reasonable to only consider second harmonic in following analysis. Considering only the first term in the Taylor expansion, and using trigonometry formulas, we can calculate the amplitude error $\Delta P_{3th}$ and phase error Δϕ of the signal as

(10) $$\Delta P_{3th}=\frac{{\left(\Delta \lambda \right)}^{3}}{48}P_{0}\left[{\left.\frac{d^{3}\alpha }{d\lambda^{3}}\right|}_{\lambda_{0}}-\sqrt{{\left(\left(1-3B^{2}\right){\left.\frac{d^{3}\alpha }{d\lambda^{3}}\right|}_{\lambda_{0}}\right)}^{2}+{\left(\frac{24}{\Delta \lambda }B{\left.\frac{d^{2}\alpha }{d\lambda^{2}}\right|}_{\lambda_{0}}\right)}^{2}}\right],$$

(11) $$\Delta \phi =\arctan\frac{24B{\left.\frac{d^{2}\alpha }{d\lambda^{2}}\right|}_{\lambda_{0}}}{\left(1-3B^{2}\right){\left.\Delta \lambda \frac{d^{3}\alpha }{d\lambda^{3}}\right|}_{\lambda_{0}}}.$$

**Figure 4:**
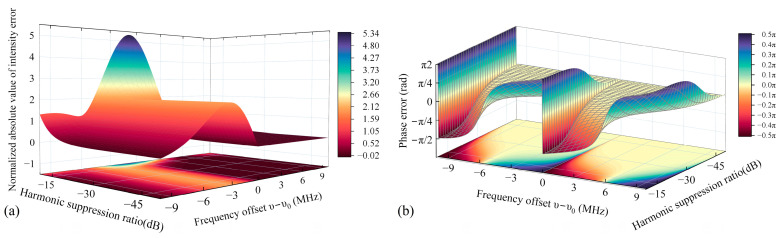
Effect of harmonic suppression ratio and frequency deviation on signal intensity error (**a**) and phase error (**b**).

Additionally, we investigated the impact of the second harmonic under varying harmonic suppression ratio (HSR), with the simulation results presented in Figure 4. As illustrated in Figure 4a, the amplitude error swiftly diminishes as the HSR increases. Interestingly, the peak amplitude error migrates toward the negative frequency deviation as the HSR rises. However, once the HSR surpasses −20 dB, further reductions in the maximum intensity error become less pronounced, a trend observed at other error points as well. Thus, excessively high HSRs are not necessarily advantageous. Similarly, the phase error is most pronounced around the center frequency, as depicted in Figure 4b. Notably, the phase error experiences a near π shift at −10 dB but drops to 10^−3^ at −20 dB. When the HSR is below −20 dB, the phase error escalates with increasing frequency deviation. Conversely, when the HSR exceeds −20 dB, the phase error remains nearly constant across all frequency deviations, barring abrupt change points.

### 2.2. Anti-Interference Algorithm

To suppress the inconsistency between the reference light and the detection light power, the instability of the light source power, and the influence of the harmonics of the modulation signal, the correction of the power ratio is adopted to cancel out the common-mode power fluctuations. For convenience, the power of the detection light and the reference light are abbreviated as the DC term and the AC term as follows

(12) $$P_{\det}=P_{1DC}+P_{1AC}\sin\left(\Omega_{m}t\right),$$

(13) $$P_{ref}=P_{2DC}+P_{2AC}\sin\left(\Omega_{m}t\right),$$

the corrected power quotient can be written as

(14) $$P_{Qcal}=\frac{P_{\det}}{P_{ref}-P_{2DC}+\frac{P_{2AC}}{P_{1AC}}P_{1DC}}\frac{P_{2AC}}{P_{1AC}}.$$

Theoretically, the value of this formula tends to 1, corresponding to the spectral signal intensity when there is no absorption by iodine molecules.

Furthermore, in order to expand the frequency locking range and enhance the signal strength, we employ the composite differential signal $S_{com}$ as the error signal.

(15) $$S_{com}=diff\left(P_{Qcal},2\right)\cdot \left[2H\left(diff\left(P_{Qcal},3\right)\right)-1\right].$$

where *H()* represents the unit step function, and *diff(f, n)* is a function for calculating the nth derivative of *f*. This signal not only significantly enhances the intensity of the signal but also eliminates the Doppler background noise.

Based on the corrected model of Wallard [18], we can derive the ratio of linear-region width (RLW) and the ratio of linear-region intensity range (RLI) between $S_{com}$ and traditional error third differential signal expressions as follows

(16) $$RLW=\frac{2\gamma }{2\sqrt{1-\sqrt{0.8}}\gamma }=3.077,$$

(17) $$RLI=\frac{5{\left(2-\sqrt{0.8}\right)}^{4}\gamma }{96\sqrt{0.8-0.8\sqrt{0.8}}}=0.1981\gamma .$$

Anti-interference frequency stabilization algorithm (Algorithm 1) based on compound differential (Pseudo-code)

**Algorithm 1.** **Anti-interference frequency stabilization algorithm**1 **Input**: Dual optical power intensity signal *P_p_*, *P_r_*.2 **Preprocessing**: Outlier detection, denoising.3 **Initialization**: Initialize the operating current, TEC working temperature, and PZT4       voltage via the host computer. Search the absorption peak position.5 **Convergence condition**: The amount of feedback is smaller than the linear region6 **Loop**:7 For time > 0 do8 Fit out the DC and AC components of *P_det_* and *P_ref_*;9 Calculate *P_Qcal_*;10 Solve the frequency deviation amount through *S_com_*;11 **End**12 **Output**: Update PZT voltage U = U + δU.

## 3. Results

The experimental schematic diagram is shown in Figure 1, and the equipment and parameters used are listed in Table 1.

**Figure 5:**
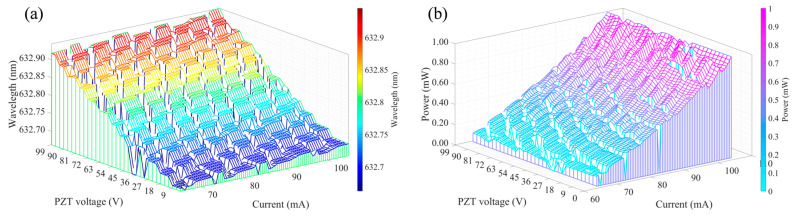
Wavelength–Voltage–Current surface (**a**); Power–Voltage–Current surface (**b**).

When the semiconductor laser is modulated by current, both its wavelength and power change. Figure 5 shows the surfaces of partial wavelength and power measured by the authors as a function of current and voltage. The wavelength was measured by a Bristol 671 series wavelength meter with an accuracy of 0.75 ppm, while the power was measured using a Thorlabs S120C probe with a resolution of 1 nW. Within the single-mode range of the laser, the wavelength exhibits an approximately linear relationship with the current, as does the power.

**Figure 6:**
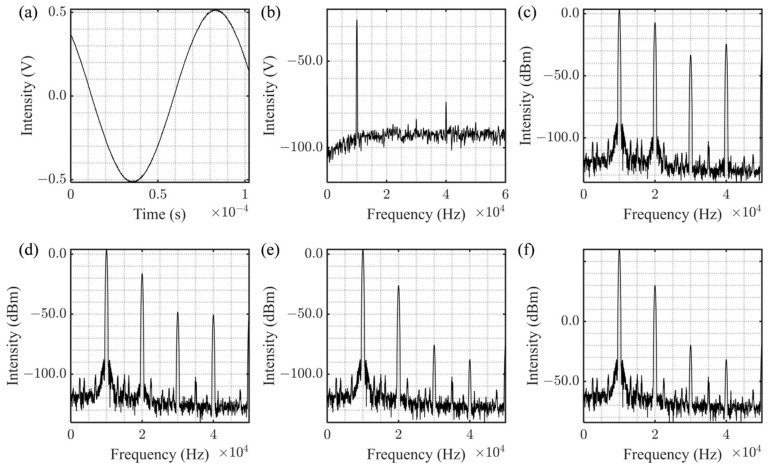
Time-domain and spectrum diagram of current modulation signal. (**a**) Signal time-domain graph; (**b**) Signal spectrum diagram; Signal spectrum diagram with HSR = 10 dB (**c**), 20 dB (**d**), 30 dB (**e**) and 30 dB with 2 Vpp (**f**).

A sine wave signal produced by a signal generator is employed to modulate the constant current source, with specific data illustrated in Figure 6. Figure 6a,b depict the time-domain and frequency-domain representations of the modulated signal, respectively. In the frequency domain, prominent harmonics are evident. By fine-tuning the amplitude of the frequency-doubled signal component, a signal with a peak-to-peak voltage (Vpp) of 1 V and varying harmonic suppression ratios is created. The corresponding spectrum diagrams are shown in Figure 6c–e, with suppression ratios of 10 dB, 20 dB, and 30 dB, respectively. Figure 6f displays a Vpp of 2 V; however, the spectrum shows no significant difference from that in Figure 6e, suggesting that the harmonic suppression ratio is not evidently correlated with the signal amplitude.

**Figure 7:**
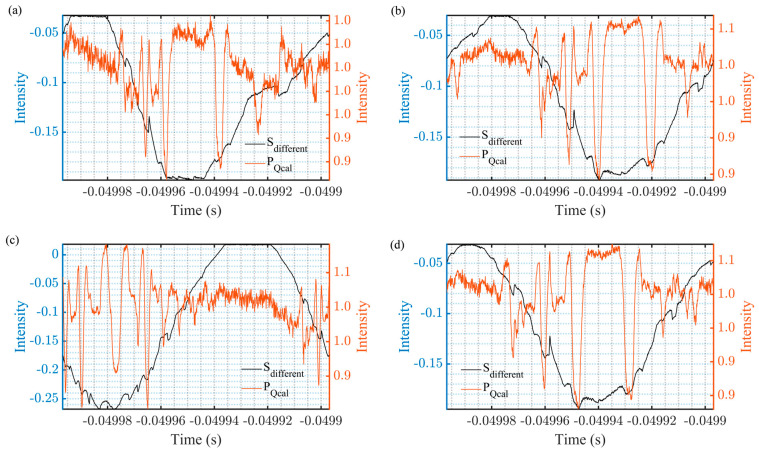
Restore the fine spectrum by using the corrected power ratio with HSR = 10 dB (**a**), 20 dB (**b**), 30 dB (**c**) and 30 dB with 2 Vpp (**d**).

The effectiveness of spectral signal restoration under various harmonic suppression ratios was evaluated using the corrected power quotient method and compared with the traditional difference demodulation method. The results are presented in Figure 7, where the conditions labeled (a–d) correspond to (c–f) in Figure 6. It is evident that the traditional difference solution exhibits significant residual sinusoidal amplitude, with the absorption signal being barely discernible. In contrast, the improved algorithm we propose yields a stable signal, which closely approximates 1 in the absence of iodine molecule absorption. At absorption wavelengths, the signal features are sharply defined, and the SNR is markedly superior to that achieved by the conventional differential algorithm.

**Figure 8:**
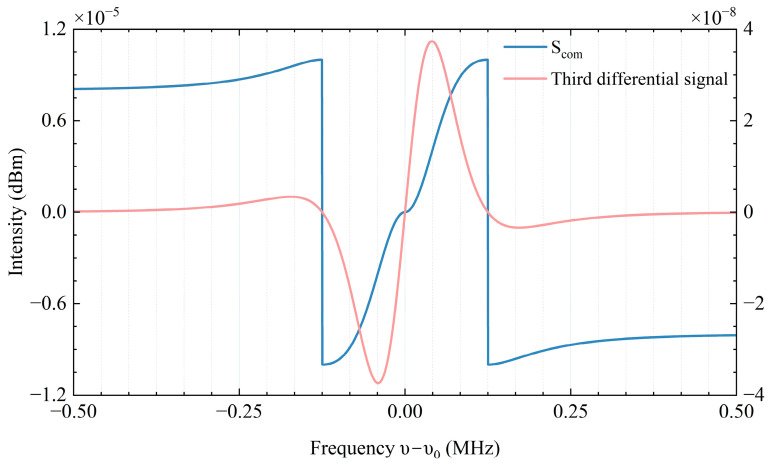
Comparison chart of composite differential signal and traditional signal.

We also conducted a preliminary assessment of the feasibility of employing the composite differentiation algorithm. As depicted in Figure 8, the error signal curves at various frequencies reveal that, in comparison to the traditional cubic differentiation error signal, the signal introduced in this study exhibits a broader monotonic interval. This indicates a more extensive frequency locking range. Additionally, the slope at the stable point (zero point) is less sensitive, and the signal demonstrates robust linear performance even at positions with significant frequency deviations.

## 4. Discussions

This section delves into the limitations of balanced detection and NICE-OHMS in the contexts examined.

The coherent optical balanced detector, which includes two symmetrical photodiodes, a differential amplifier, and a beam splitter, functions based on coherent mixing and differential processing. The signal and local oscillator (reference) lights are divided into two paths by the beam splitter and directed into the photodiodes. Due to the π  phase difference from the beam splitter, the light paths create opposite-phase interference patterns on the detector surface, generating photocurrents of equal magnitude but opposite direction. The differential amplifier then subtracts these signals to produce a differential signal that contains only phase and frequency information. When considering the power noise of the light source itself, the final demodulated voltage signal obtained is

(18) $$U\propto 2\sqrt{P_{1DC}P_{2DC}+\left(P_{1DC}P_{2AC}+P_{2DC}P_{1AC}\right)\sin\left(\Omega_{m}t\right)+P_{1AC}P_{2AC}\sin^{2}\left(\Omega_{m}t\right)}.$$

Clearly, the balanced detection signal now contains interference from the modulated signal, making it impossible to directly recover the power variations of the probe signal.

NICE-OHMS is a powerful technique for detecting molecular compounds in the gas phase. It combines frequency modulation spectroscopy (FMS) to reduce noise and cavity enhancement to prolong the interaction length between light and the sample [20]. The FMS signal is obtained by demodulating the detector signal at the modulation frequency at a given detection phase $\theta_{fm}$. Because the power of the semiconductor laser is not constant during FMS, the DC signal expanded into a Bessel series ($J_{m}\left(\beta \right)$) must be corrected to

(19) $$\begin{array}{c} S_{T}^{fm}\left(v_{d},\theta_{fm}\right)=\eta_{fm}\left(P_{0DC}+P_{0AC}\sin\left(\Omega_{m}t+\theta_{0}\right)\right)J_{0}\left(\beta \right)J_{1}\left(\beta \right)\times \\ \;\;\;\;\;\;\;\;\;\;\;\;\;\left\{\left[\phi_{-1}\left(v_{d}\right)-2\phi_{0}\left(v_{d}\right)+\phi_{1}\left(v_{d}\right)\right]\cos\theta_{fm}+\left[\delta_{-1}\left(v_{d}\right)-\delta_{1}\left(v_{d}\right)\right]\sin\theta_{fm}\right\}. \end{array}$$

where the phase of the electric field is modulated at a radio frequency $v_{m}$, with an amplitude β, $\delta_{j}\left(\nu_{c}\right)$ and $\phi_{j}\;\left(\nu_{c}\right)$ are the amplitude attenuation and the optical phase shift of the electrical field, $\nu_{d}=\;v_{0}-v_{c}\;$ is the detuning of the laser frequency from the transition center frequency $\;v_{0}$, $P_{0}$ is the optical power incident on the detector and $\;\eta_{fm}$ is an instrumentation factor, $\theta_{fm}$ is detection phase. Evidently, the presence of the same-frequency component in the power leads to the generation of additional signals, such as the double frequency, upon demodulation, thereby yielding erroneous information.

**Figure 9:**
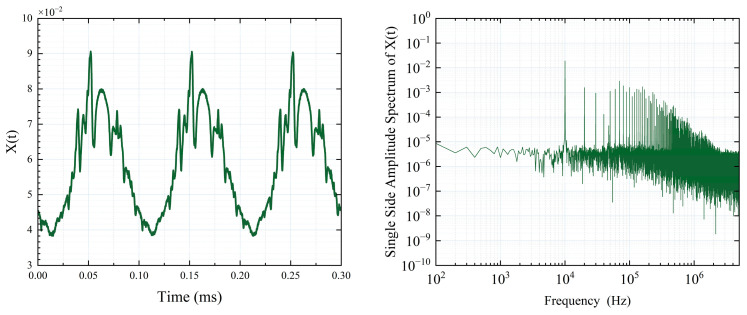
Time-domain graph of the spectral signal (**left**) and its spectrum (**right**).

Moreover, ensuring consistent intensities between the detection light and the reference light poses a significant challenge. Additionally, applying NICE-OHMS technology with an FP cavity in environmental detection scenarios is fraught with difficulties. Therefore, we conclude that while balanced detection can suppress common-mode noise introduced by environmental interference, it cannot eliminate the power noise introduced by the light source due to modulation. NICE-OHMS can enhance resistance to environmental noise by capitalizing on the characteristics of the FP cavity when the light source power remains constant, but it is not suitable for the specific scenario outlined in this paper. Furthermore, balanced detection necessitates the use of a low-pass filter to eliminate AC components, a process that typically results in the loss of target information. As illustrated in Figure 9, low-pass filtering leads to the attenuation of most high-frequency spectral components.

## 5. Conclusions

This study has developed a signal processing method and a feedback control method based on spectral signal sensing. Utilizing the corrected power quotient, the processing method exhibits insensitivity to power noise and achieves a substantial enhancement in the signal-to-noise ratio. The research offers significant guidance for spectral signal sensing analysis and monitoring in engineering applications. Additionally, while the feedback control method discussed in this text has theoretical advantages such as a wide locking range and good linearity, further work is required for verification.

## Figures and Tables

**Table 1:** Table Experimental parameters settings.

Equipment	Parameter	Brand
Laser	-	LDPD-INC
Constant current source	89.4 mA	Thorlabs LDC202C
Temperature control device	10 kΩ	Thorlabs TED200C
Detector	20 dB/30 dB *	Thorlabs PDA36A2
Power meter	-	Thorlabs PM400 & S120C
Signal generator	-	Moku: pro
spectrum analyzer	-	Moku: pro
Wavelength meter	-	Bristol

* Reference light (20 uW) has a 20 dB gain, and reference light (2.7 uW) has a 30 dB gain.

## Data Availability

The data related to this study can be requested from the corresponding author.

## Abbreviations

The following abbreviations are used in this manuscript:

BS	Beam Splitter
CCC	Current Control Circuit
DBR	Distributed Bragg Reflector
DFB	Distributed Feedback
FOC	Fiber Optical Coupler
FPGA	Field Programmable Gate Array
FMS	Frequency Modulation Spectroscopy
HWP	Half-wave Plate
HSR	Harmonic Suppression Ratio
MHN	Modulation harmonic noise
NICE-OHMS	Noise-Immune Cavity-Enhanced Optical Heterodyne Molecular Spectroscopy
Vpp	Peak-to-peak Voltage
PD	Photodetector
PZT	Piezoelectric Transducer
PBS	Polarization Beam Splitter
POL	Polarizer
PMN	Power Modulation Noise
PLC	Programmable Logic Controller
PCC	PZT Control Circuit
RLI	Ratio of Linear-region Intensity Range
RLW	Ratio of Linear-Region Width
SAS	Saturated Absorption Spectroscopy
SNR	Signal-to-Noise Ratio
TCC	Temperature Control Circuit
TEC	Thermoelectric Cooler
